# *N*′-(But-2-enyl­idene)­iso­nicotino­hydrazide

**DOI:** 10.1107/S1600536808033266

**Published:** 2008-10-22

**Authors:** Zhi-Gang Yin, Shu-Mian Li, Heng-Yu Qian, Yu-Zhen Chen

**Affiliations:** a Key Laboratory of Surface and Interface Science of Henan, School of Materials and Chemical Engineering, Zhengzhou University of Light Industry, Zhengzhou 450002, People’s Republic of China; b School of Chemistry and Chemical Engineering, Henan University of Technology, Zhengzhou 450052, People’s Republic of China

## Abstract

In the title Schiff base compound, C_10_H_11_N_3_O, the pyridine ring is twisted with respect to the mean plane containing the hydrazine chain, making a dihedral angle of 31.40 (9)°. The NH group inter­acts with the N atom of the pyridine ring through N—H⋯N hydrogen bonds to build up a zigzag chain developing parallel to the (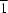
01) plane.

## Related literature

For general background, see: Kahwa *et al.* (1986 ▶); Santos *et al.* (2001 ▶). 
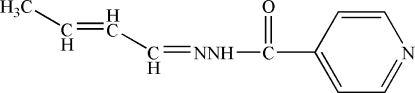


## Experimental

### 

#### Crystal data

C_10_H_11_N_3_O
*M* *_r_* = 189.22
Monoclinic, 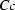

*a* = 9.5779 (8) Å
*b* = 12.6191 (11) Å
*c* = 9.2095 (8) Å
β = 113.511 (1)°
*V* = 1020.70 (15) Å^3^
*Z* = 4
Mo *K*α radiation
μ = 0.08 mm^−1^
*T* = 293 (2) K
0.25 × 0.23 × 0.18 mm

#### Data collection

Bruker SMART CCD area-detector diffractometer
Absorption correction: multi-scan (*SADABS*; Bruker, 1998 ▶) *T*_min_ = 0.969, *T*_max_ = 0.974
4639 measured reflections
1264 independent reflections
1225 reflections with *I* > 2σ(*I*)
*R*_int_ = 0.012

#### Refinement

*R*[*F* ^2^ > 2σ(*F* ^2^)] = 0.034
*wR*(*F* ^2^) = 0.101
*S* = 1.08
1264 reflections
128 parameters
2 restraints
H-atom parameters constrained
Δρ_max_ = 0.21 e Å^−3^
Δρ_min_ = −0.12 e Å^−3^

### 

Data collection: *SMART* (Bruker, 1998 ▶); cell refinement: *SAINT* (Bruker, 1998 ▶); data reduction: *SAINT*; program(s) used to solve structure: *SHELXS97* (Sheldrick, 2008 ▶); program(s) used to refine structure: *SHELXL97* (Sheldrick, 2008 ▶); molecular graphics: *ORTEPIII* (Burnett & Johnson, 1996 ▶), *ORTEP-3 for Windows* (Farrugia, 1997 ▶) and *PLATON* (Spek, 2003 ▶).; software used to prepare material for publication: *SHELXL97*.

## Supplementary Material

Crystal structure: contains datablocks global, I. DOI: 10.1107/S1600536808033266/dn2392sup1.cif

Structure factors: contains datablocks I. DOI: 10.1107/S1600536808033266/dn2392Isup2.hkl

Additional supplementary materials:  crystallographic information; 3D view; checkCIF report

## Figures and Tables

**Table 1:** Hydrogen-bond geometry (Å, °)

*D*—H⋯*A*	*D*—H	H⋯*A*	*D*⋯*A*	*D*—H⋯*A*
N2—H2⋯N3^i^	0.86	2.17	2.991 (2)	160

Symmetry code: (i) 
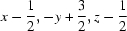
.

**Fig. 1.**
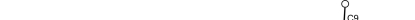
Molecular view of (I) with the atom-labeling scheme. Displacement ellipsoids are drawn at the 30% probability level. H atoms are presented as small spheres of arbitrary radii.

**Fig. 2.**
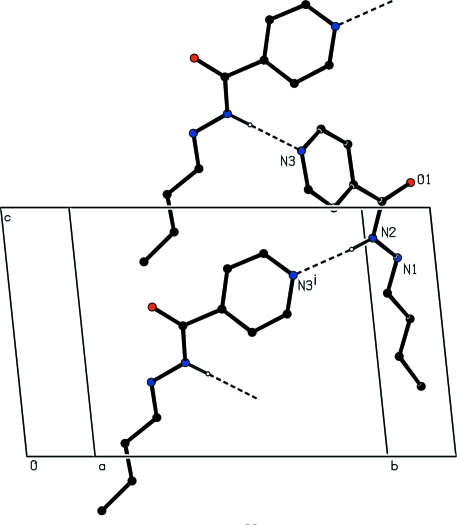
Partial packing view showing the formation of the zig-zag chain through N-H···N hydrogen bonds which are shown as dashed lines. H atoms not involved in hydrogen bondings have been omitted for clarity.[Symmetry code: (i) x-1/2, -y+3/2, z-1/2]

## supplementary crystallographic information

### Comment

The chemistry of Schiff bases has attracted a great deal of interest in recent years. These compounds play an important role in the development of various proteins and enzymes(Kahwa *et al.*, 1986; Santos *et al.*, 2001). As part of our interest in the study of the coordination chemistry of Schiff bases, we have synthesized the title compound (I) and reported its cyrstal structure.

In the title compound. the pyridine ring is twisted with respect to the mean plane containing the hydrazine chain making a dihedral angle of 31.40 (9)° (Fig. 1). The NH interacts with the nitrogen atom of the pyridine ring through N-H···N hydrogen bond to build up a zig-zag chain developing parallel to the (-1 0 1) plane (Table 1, Fig. 2).

### Experimental

Pyridine-4-carboxylic acid hydrazide (1 mmol, 0.137 g) was dissolved in anhydrous methanol, H_2_SO_4_ (98% 0.5 ml) was added to this, the mixture was stirred for several minitutes at 351 K, 3,4-dichlorobenzyaldehyde (1 mmol 0.070 g) in methanol (8 ml) was added dropwise and the mixture was stirred at refluxing temperature for 2 h. The product was isolated and recrystallized in dichloromethane, brown single crystals of (I) was obtained after 5 d.

### Refinement

H atoms were placed in calculated position and treated as riding with C—H= 0.93Å (aromatic), 0.96Å(methyl) and N-H= 0.86\%A with *U*_iso_(H)=1.2*U*_eq_(C,N) or *U*_iso_(H)=1.5*U*_eq_(methyl).

In the absence of significant anomalous scattering, the absolute structure could not be reliably determined and then the Friedel pairs were merged and any references to the Flack parameter were removed.

### Crystal data

**Table:**

C_10_H_11_N_3_O	*F*(000) = 400
*M**_r_* = 189.22	*D*_x_ = 1.231 Mg m^−^^3^
Monoclinic, *C**c*	Mo *K*α radiation, λ = 0.71073 Å
Hall symbol: C -2yc	Cell parameters from 2994 reflections
*a* = 9.5779 (8) Å	θ = 2.4–23.8°
*b* = 12.6191 (11) Å	µ = 0.08 mm^−^^1^
*c* = 9.2095 (8) Å	*T* = 293 K
β = 113.511 (1)°	Block, brown
*V* = 1020.70 (15) Å^3^	0.25 × 0.23 × 0.18 mm
*Z* = 4	

### Data collection

**Table:**

Bruker SMART CCD area-detector diffractometer	1264 independent reflections
Radiation source: fine-focus sealed tube	1225 reflections with *I* > 2σ(*I*)
graphite	*R*_int_ = 0.012
ω scans	θ_max_ = 28.3°, θ_min_ = 2.8°
Absorption correction: multi-scan (SADABS; Bruker, 1998)	*h* = −12→12
*T*_min_ = 0.969, *T*_max_ = 0.974	*k* = −16→16
4639 measured reflections	*l* = −12→12

### Refinement

**Table:**

Refinement on *F*^2^	Primary atom site location: structure-invariant direct methods
Least-squares matrix: full	Secondary atom site location: difference Fourier map
*R*[*F*^2^ > 2σ(*F*^2^)] = 0.034	Hydrogen site location: inferred from neighbouring sites
*wR*(*F*^2^) = 0.101	H-atom parameters constrained
*S* = 1.08	*w* = 1/[σ^2^(*F*_o_^2^) + (0.0744*P*)^2^ + 0.0728*P*] where *P* = (*F*_o_^2^ + 2*F*_c_^2^)/3
1264 reflections	(Δ/σ)_max_ < 0.001
128 parameters	Δρ_max_ = 0.21 e Å^−^^3^
2 restraints	Δρ_min_ = −0.12 e Å^−^^3^

### Special details

**Table:**

Geometry. All esds (except the esd in the dihedral angle between two l.s. planes) are estimated using the full covariance matrix. The cell esds are taken into account individually in the estimation of esds in distances, angles and torsion angles; correlations between esds in cell parameters are only used when they are defined by crystal symmetry. An approximate (isotropic) treatment of cell esds is used for estimating esds involving l.s. planes.
Refinement. Refinement of *F*^2^ against ALL reflections. The weighted *R*-factor *wR* and goodness of fit *S* are based on *F*^2^, conventional *R*-factors *R* are based on *F*, with *F* set to zero for negative *F*^2^. The threshold expression of *F*^2^ > σ(*F*^2^) is used only for calculating *R*-factors(gt) *etc*. and is not relevant to the choice of reflections for refinement. *R*-factors based on *F*^2^ are statistically about twice as large as those based on *F*, and *R*- factors based on ALL data will be even larger.

### Fractional atomic coordinates and isotropic or equivalent isotropic displacement parameters (Å^2^)

**Table:**

	*x*	*y*	*z*	*U*_iso_*/*U*_eq_	
N1	0.12133 (17)	1.06196 (12)	0.79852 (17)	0.0441 (3)	
N2	0.21940 (16)	0.98057 (11)	0.87720 (16)	0.0398 (3)	
H2	0.2126	0.9200	0.8322	0.048*	
N3	0.6220 (2)	0.73212 (13)	1.2278 (2)	0.0512 (4)	
O1	0.33989 (17)	1.07888 (10)	1.10035 (17)	0.0542 (4)	
C1	−0.2651 (3)	1.1633 (3)	0.2819 (3)	0.0800 (8)	
H1A	−0.2643	1.2313	0.3292	0.120*	
H1B	−0.3661	1.1344	0.2431	0.120*	
H1C	−0.2337	1.1712	0.1957	0.120*	
C2	−0.1590 (3)	1.0910 (2)	0.4019 (3)	0.0625 (5)	
H2A	−0.1514	1.0222	0.3696	0.075*	
C3	−0.0728 (2)	1.11582 (19)	0.5528 (2)	0.0546 (5)	
H3	−0.0819	1.1824	0.5913	0.066*	
C4	0.0338 (2)	1.04042 (16)	0.6560 (2)	0.0475 (4)	
H4	0.0388	0.9732	0.6170	0.057*	
C5	0.32562 (18)	0.99674 (12)	1.02479 (18)	0.0366 (3)	
C6	0.42972 (17)	0.90326 (12)	1.09198 (17)	0.0362 (3)	
C7	0.4905 (3)	0.88762 (17)	1.2543 (2)	0.0529 (5)	
H7	0.4696	0.9347	1.3208	0.064*	
C8	0.5828 (3)	0.80039 (19)	1.3153 (2)	0.0584 (5)	
H8	0.6198	0.7887	1.4239	0.070*	
C9	0.5679 (2)	0.75022 (15)	1.0721 (2)	0.0477 (4)	
H9	0.5965	0.7046	1.0096	0.057*	
C10	0.47069 (19)	0.83399 (13)	0.99866 (19)	0.0411 (3)	
H10	0.4341	0.8433	0.8896	0.049*	

### Atomic displacement parameters (Å^2^)

**Table:**

	*U*^11^	*U*^22^	*U*^33^	*U*^12^	*U*^13^	*U*^23^
N1	0.0439 (8)	0.0368 (7)	0.0431 (8)	0.0086 (6)	0.0081 (6)	0.0041 (6)
N2	0.0428 (7)	0.0303 (6)	0.0381 (7)	0.0045 (5)	0.0075 (5)	−0.0011 (5)
N3	0.0596 (9)	0.0426 (8)	0.0425 (8)	0.0149 (7)	0.0108 (7)	0.0071 (6)
O1	0.0604 (8)	0.0401 (7)	0.0468 (7)	0.0119 (6)	0.0055 (6)	−0.0113 (5)
C1	0.0650 (15)	0.099 (2)	0.0570 (13)	0.0146 (14)	0.0046 (11)	0.0212 (14)
C2	0.0564 (11)	0.0683 (14)	0.0532 (12)	0.0083 (10)	0.0116 (9)	0.0077 (10)
C3	0.0481 (9)	0.0564 (11)	0.0491 (10)	0.0114 (8)	0.0086 (8)	0.0117 (8)
C4	0.0453 (9)	0.0441 (9)	0.0445 (9)	0.0048 (7)	0.0088 (7)	0.0027 (7)
C5	0.0387 (7)	0.0320 (7)	0.0351 (7)	0.0046 (5)	0.0104 (6)	−0.0001 (5)
C6	0.0381 (7)	0.0322 (7)	0.0338 (7)	0.0025 (5)	0.0096 (6)	−0.0003 (6)
C7	0.0662 (12)	0.0525 (11)	0.0334 (8)	0.0194 (9)	0.0128 (8)	−0.0008 (7)
C8	0.0720 (12)	0.0601 (12)	0.0334 (8)	0.0204 (10)	0.0108 (8)	0.0074 (8)
C9	0.0585 (10)	0.0395 (8)	0.0423 (8)	0.0146 (7)	0.0173 (8)	0.0013 (6)
C10	0.0494 (8)	0.0366 (8)	0.0332 (7)	0.0088 (6)	0.0122 (6)	0.0014 (6)

### Geometric parameters (Å, °)

**Table:**

N1—C4	1.273 (2)	C3—C4	1.441 (3)
N1—N2	1.3853 (18)	C3—H3	0.9300
N2—C5	1.349 (2)	C4—H4	0.9300
N2—H2	0.8600	C5—C6	1.509 (2)
N3—C8	1.332 (3)	C6—C7	1.385 (2)
N3—C9	1.335 (2)	C6—C10	1.388 (2)
O1—C5	1.225 (2)	C7—C8	1.383 (3)
C1—C2	1.480 (3)	C7—H7	0.9300
C1—H1A	0.9600	C8—H8	0.9300
C1—H1B	0.9600	C9—C10	1.392 (2)
C1—H1C	0.9600	C9—H9	0.9300
C2—C3	1.340 (3)	C10—H10	0.9300
C2—H2A	0.9300		
			
C4—N1—N2	114.28 (15)	C3—C4—H4	118.6
C5—N2—N1	119.75 (13)	O1—C5—N2	124.64 (15)
C5—N2—H2	120.1	O1—C5—C6	121.51 (15)
N1—N2—H2	120.1	N2—C5—C6	113.84 (13)
C8—N3—C9	117.17 (16)	C7—C6—C10	118.54 (14)
C2—C1—H1A	109.5	C7—C6—C5	118.58 (14)
C2—C1—H1B	109.5	C10—C6—C5	122.85 (14)
H1A—C1—H1B	109.5	C8—C7—C6	118.48 (16)
C2—C1—H1C	109.5	C8—C7—H7	120.8
H1A—C1—H1C	109.5	C6—C7—H7	120.8
H1B—C1—H1C	109.5	N3—C8—C7	123.92 (17)
C3—C2—C1	125.9 (2)	N3—C8—H8	118.0
C3—C2—H2A	117.1	C7—C8—H8	118.0
C1—C2—H2A	117.1	N3—C9—C10	123.32 (16)
C2—C3—C4	120.8 (2)	N3—C9—H9	118.3
C2—C3—H3	119.6	C10—C9—H9	118.3
C4—C3—H3	119.6	C6—C10—C9	118.48 (15)
N1—C4—C3	122.76 (18)	C6—C10—H10	120.8
N1—C4—H4	118.6	C9—C10—H10	120.8

### Hydrogen-bond geometry (Å, °)

**Table:**

*D*—H···*A*	*D*—H	H···*A*	*D*···*A*	*D*—H···*A*
N2—H2···N3^i^	0.86	2.17	2.991 (2)	160

Symmetry codes: (i) *x*−1/2, −*y*+3/2, *z*−1/2.
